# Fasting-Mimicking Diet Inhibits Autophagy and Synergizes with Chemotherapy to Promote T-Cell-Dependent Leukemia-Free Survival

**DOI:** 10.3390/cancers15245870

**Published:** 2023-12-16

**Authors:** Roberta Buono, Jonathan Tucci, Raffaello Cutri, Novella Guidi, Serghei Mangul, Franca Raucci, Matteo Pellegrini, Steven D. Mittelman, Valter D. Longo

**Affiliations:** 1 Department of Biological Sciences, Longevity Institute, School of Gerontology, University of Southern California, 3715 McClintock Avenue, Los Angeles, CA 90089, USA; 2 Department of Molecular Biology and Biochemistry, University of California, Irvine, CA 92697, USA; 3 Center for Endocrinology, Diabetes & Metabolism, Children’s Hospital Los Angeles, 4650 Sunset Blvd, Los Angeles, CA 90027, USA; 4 Department of Computer Science, University of California Los Angeles, 580 Portola Plaza, Los Angeles, CA 90095, USA; 5 Institute for Quantitative and Computational Biosciences, Boyer Hall, 611 Charles Young Drive, University of California Los Angeles, Los Angeles, CA 90095, USA; 6 IFOM AIRC Institute of Molecular Oncology, Via Adamello 16, 20139 Milan, Italy; 7 Department of Molecular, Cell and Developmental Biology, University of California Los Angeles, 801 Hilgard Avenue, Los Angeles, CA 90095, USA; 8 Division of Pediatric Endocrinology, UCLA Mattel Children’s Hospital, 10833 Le Conte Avenue, MDCC 22-315, Los Angeles, CA 90095, USA; 9 Eli and Edythe Broad Center for Regenerative Medicine and Stem Cell Research at USC, Keck School of Medicine, University of Southern California, Los Angeles, CA 90033, USA

**Keywords:** Leukemia, pre-B-ALL, fasting-mimicking diet, autophagy, cancer treatment

## Abstract

**Simple Summary:**

Despite the advances in the treatment of pre-B-ALL leukemia in children, adult pre-B-ALL continues to represent a major challenge. This work focuses on the use of differential responses to fasting conditions between normal and cancer cells to achieve cancer-free survival. We show that a fasting-mimicking diet in combination with vincristine causes a synergistic increase in the toxicity to pre-B-ALL cells resulting in high cancer cell death. While fasting is not sufficient to promote cancer-free survival, the combination of fasting/FMD and vincristine promotes autophagy inhibition, which is at the center of the high toxicity phenotype specific to leukemia cells, possibly through a mechanism involving immune cells.

**Abstract:**

Fasting mimicking diets (FMDs) are effective in the treatment of many solid tumors in mouse models, but their effect on hematologic malignancies is poorly understood, particularly in combination with standard therapies. Here we show that cycles of a 3-day FMD given to high-fat-diet-fed mice once a week increased the efficacy of vincristine to improve survival from BCR-ABL B acute lymphoblastic leukemia (ALL). In mice fed a standard diet, FMD cycles in combination with vincristine promoted cancer-free survival. RNA seq and protein assays revealed a vincristine-dependent decrease in the expression of multiple autophagy markers, which was exacerbated by the fasting/FMD conditions. The autophagy inhibitor chloroquine could substitute for fasting/FMD to promote cancer-free survival in combination with vincristine. In vitro, targeted inhibition of autophagy genes *ULK1* and *ATG9a* strongly potentiated vincristine’s toxicity. Moreover, anti-CD8 antibodies reversed the effects of vincristine plus fasting/FMD in promoting leukemia-free survival in mice, indicating a central role of the immune system in this response. Thus, the inhibition of autophagy and enhancement of immune responses appear to be mediators of the fasting/FMD-dependent cancer-free survival in ALL mice.

## 1. Introduction

Pre-B cell acute lymphoblastic leukemia (ALL) is the most common childhood cancer but can also occur in older adults. It is a hematologic malignancy characterized by impaired differentiation and aggressive proliferation of clonal lymphoblasts in the bone marrow, spleen and blood [1,2]. The standard of care for B-ALL is chemotherapy, which includes vincristine, cyclophosphamide, anthracyclines, corticosteroids, L-asparaginase and other drugs [3]. The overall cure rate is about 90% in children but only 50% in adults, for whom the relapse occurrence is high due to drug resistance [2]. There is still no consensus regarding the best treatment approach for newly diagnosed adults and especially older patients. 

It is becoming increasingly recognized that weight and nutritional status can impact cancer survival and that obesity is a risk factor for poor outcomes not only for B-ALL but also for other cancers [4,5,6]. Using a murine model of diet-induced obesity, obese mice with syngeneic BCR-ABL B-ALL were shown to have poorer response to chemotherapy than control mice [7,8,9]. Clinical studies have shown similar negative effects of obesity on cancer mortality [10].

We and other groups have previously demonstrated that periodic fasting or FMDs reduce chemotherapy side effects, improve cancer treatment efficacy and delay cancer progression in mice [11,12,13]. Here, we have investigated the effects of FMD cycles in combination with or without chemotherapy (vincristine) in both obese and normal weight syngeneic in vivo mouse models as well as in vitro models for mouse and human B-ALL. 

## 2. Materials and Methods

### 2.1. Cell Culture and Treatment

BCR-ABL syngeneic leukemia cells (“8093 cells”) were generated using BCR/ABL transgenic mouse [16] M-ALL were cultured at a density of 2.5 × 10^5^ cells/mL in a standard condition McCoy’s 5A media supplemented with 10% FBS, murine IL-3, beta-mercapto ethanol and gentamycin or in the STS condition (same as above but with 0.5 gr/liter of Glucose; 2% FBS).

Human leukemia cells H-ALL (BV173) were obtained from ATCC and were cultured in RPMI media supplemented with 10% FBS and gentamycin or in the STS condition.

M-ALL and H-ALL were treated for 24 h or 48 h with or without VC 5nM (Sigma Aldrich, Saint Louis, MO, USA Cat#V8388). 

### 2.2. Cell Viability

Cells viability was measured by Mini Automated Cell Counter Moxi (Orflo, Ketchum, ID, USA) or by Trypan Blue exclusion dye (Corning, Glendale, AZ, USA Cat# MT25900CI).

### 2.3. ULK1 and ATG9a Silencing

Cells were seeded at 60–80% of confluence and then transfected for 48 h with 30 pM ULK1 and ATG9a siRNA (Life Technologies, Carlsbad, CA, USA ID: 65268, 125425) using Lipofectamine RNAiMAx Reagent (Thermo Fisher Scientific, Waltham, MA, USA Cat#13778100) according to the manufacturer’s instructions.

### 2.4. LDH Assay

Cell cytotoxicity was measured using the colorimetric CytoTox 96^®^ Non-Radioactive Cytotoxicity Assay (Promega, Madison, WI, USA, Cat# G1780) following the manufacturer’s instructions.

### 2.5. CYTO-ID Staining

Autophagy was measured by the CYTO-ID^®^ Autophagy detection kit (Enzo Life Sciences, Farmingdale, NY, USA Cat# ENZ-51031-0050) according to the manufacturer’s protocols.

### 2.6. FACS Analyses

Flow cytometry analyses of mouse BM and SP GFP^+^ leukemia cells were performed to assess the engraftment. Flow cytometry staining of BM, spleen and blood were performed using APC-CD3 (Thermo Fisher Scientific Cat# 17-0032-82), PerCP-eFluor710-CD8 (Thermo Fisher Scientific Cat# 46-0081-80), PE-CD4 (Thermo Fisher Scientific Cat# 12-0041-85), APC-eFluor780-CD25 (Thermo Fisher Scientific Cat# 47-0251-82) and PE-Cy7-PD1 antibodies (Thermo Fisher Scientific Cat# 25-9985-82). FlowJo 10 (Becton Dickinson, Franklin Lakes, NJ, USA) was used to analyze data and to prepare figures.

### 2.7. Annexin V Staining

BCR-ABL syngeneic leukemia cells and BV173 were stained with eFLuor780 Fixable Viability Dye (Thermo Fisher Scientific Cat# 65-0865) and PE-Cy7 Annexin V (Thermo Fisher Scientific Cat# 88-8103-72) according to the manufacturer’s instructions. Analyses were performed with BD FACS diva on LSR II (Becton Dickinson, Franklin Lakes, NJ, USA).

### 2.8. Western Blotting

Total cell lysates were prepared using the RIPA buffer (Thermo Fisher Scientific Cat# 89900) according to the manufacturer’s instructions. Protein concentration was measured with the BCA assay (Thermo Fisher Scientific Cat#23227). Equal amounts of protein (30 µg) were heat-denaturized in a lane marker-reducing sample buffer (Thermo Fisher Scientific Cat#39000), resolved by SDS-PAGE using Novex 4–20% Tris-Glycine MiniProtein Gels (Thermo Fisher Scientific) and transferred to PVDF membranes (Millipore, Darmstadt, Germany). The filters were blocked in 5% BSA for 1 h at room temperature and then incubated O.N at 4° with a primary antibody directed against cleaved caspase 3 (1:1000), phosphorylated p53 (1:1000), beclin1 (1:1000), p62 (1:1000), LC3B (1:1000), ULK1 (1:1000), vinculin (1:2000) (Cell Signaling, Danvers, MA, USA, rabbit mAb #9664, rabbit mAb #9284, rabbit mAb #3495, rabbit mAb #5114, rabbit mAb #2775, rabbit mAb #8054, rabbit mAb #18799), ATG9 (1:1000) (GeneTex, Irvine, CA, USA, rabbit mAb # GTX128427) and tubulin (1:2500) (Millipore, Burlington, MA, USA, #05-661). 

Peroxidase-conjugated IgG (Santa Cruz, CA, USA) was used as a secondary antibody.

ImageJ software version 1.52a was used to analyze western blot data.

### 2.9. RNA-seq Library Preparation and Data Analysis

(Supplementary methods): RNAseq data are deposited in the GEO database GSE212918. 

### 2.10. In Vivo B-ALL Model

All animal protocols were approved by the Institutional Animal Care and Use Committee (IACUC) of the University of Southern California. All mice were maintained in a pathogen-free environment and housed in clear shoebox cages in groups of five animals per cage with a constant temperature and humidity, a 12 h/12 h light/dark cycle and unlimited access to water.

Obese model: Diet-induced obese C57BL/6J mice (Jackson Laboratories, Bar Harbor, ME, USA, Cat# 380050) were weaned onto 60% calories from fat diet (Research Diets, New Brunswick, NJ, USA, D12492) and maintained on the diet until dietary intervention. At 20 weeks of age, mice were injected retro-orbitally with 1 × 10^4^ GFP-expressing BCR-ABL syngeneic leukemia cancer cells. One week later, mice were divided into 4 groups: HFD + vehicle (HFD *n* = 5), HFD + FMD (*n* = 5) 4 cycles + vehicle, HFD + chemo drugs (vincristine (HFD + VC *n* = 5)) I.P. 0.5 mg/kg once a week and HFD + FMD +VC (*n* = 5) (VC Sigma Aldrich, St. Louis, MO, USA, Cat#V8388).

Normal diet model: 50 C57BL/6J mice (Jackson Laboratories Cat# 000664) (20 weeks old) were injected as described above. One week later, the mice were divided into 4 groups: ad lib+ vehicle (AL *n* = 10), FMD (*n* = 12) 4 cycles + vehicle, ad lib+ vincristine (AL + VC *n* = 14) I.P. 0.5 mg/kg once a week and FMD +VC (*n* = 14).

### 2.11. Mouse FMD Diet

The mouse version of the FMD was a 3-day regimen [14,15] (Table S1 and supplementary methods). 

### 2.12. Vincristine and Chloroquine in an In Vivo Model of BCR-ABL B-ALL

40 mice C57BL/6J (20 weeks old) were injected with GFP-expressing BCR-ABL syngeneic leukemia cancer cells. One week later after tumor injection, they were divided into 5 groups: ad lib+ vehicle (AL), ad lib+ chloroquine once a week I.P. 50 mg/kg/day (AL + CQ *n* = 8), fasting-mimicking diet (FMD + CQ *n* = 8) 4 cycles + CQ, ad lib+ VC + CQ once a week (AL + VC + CQ *n* = 8) and FMD +VC + CQ (*n* = 8) (CQ Sigma Aldrich Cat# PHR1258).

### 2.13. CD8+ Cells In Vivo Depletion

Complete depletion of CD8+ CTL was achieved by intraperitoneal administration of neutralizing monoclonal antibody (αCD8; clone YTS 169.4 BioXCell Lebanon, NH, USA, Cat# BP0117) or rat IgG (BioXCell, Cat# BP0090) every 4 days after 1 week of tumor implantation. The depletion of circulating CD8+ CTL over time was confirmed by FACS analysis.

### 2.14. Statistics and Experimental Design

For statistical significance of the differences between the means of the two groups, we used two-tailed Student’s *t*-tests. The statistical significance of differences among multiple groups (≥3) was calculated by performing ANOVA multiple comparisons of the means for each group. The survival rates of the two groups were analyzed using a log–rank test and were considered to be statistically significant if *p* < 0.05. 

No samples or animals were excluded from analysis, and sample size estimates were not used. Animals were randomly assigned to groups. Studies were not conducted blinded.

## 3. Results

### 3.1. FMD Cycles Promote Cancer-Free Survival in Obese Leukemic Mice in Combination with Vincristine

We investigated whether FMD cycles, in combination with vincristine therapy, could improve the outcome of BCR-ABL B-ALL in an obese murine model.

**Figure 1:**
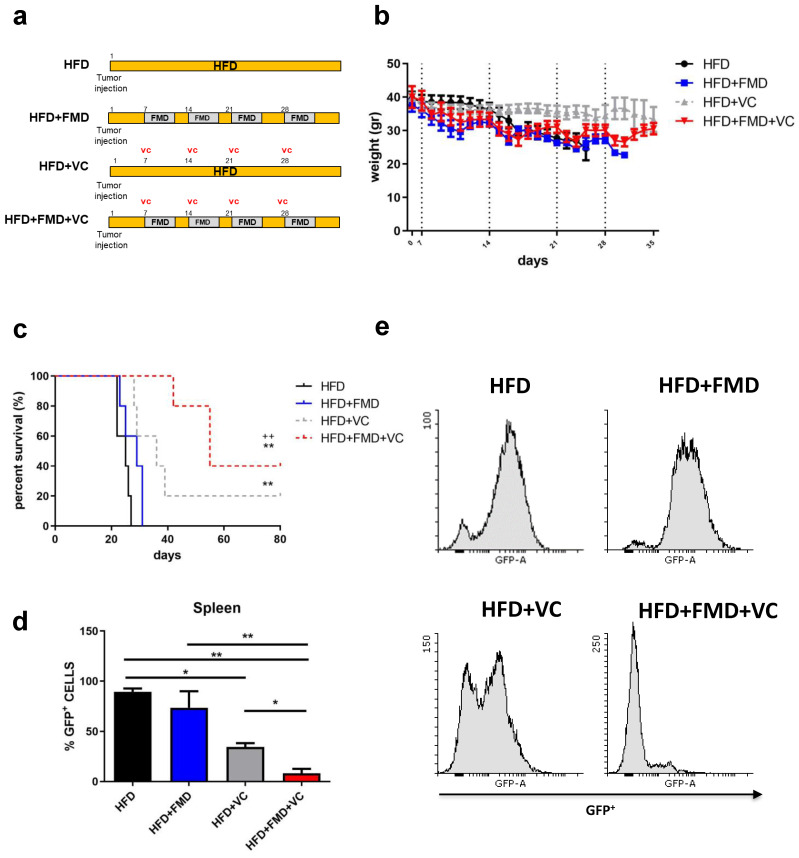
FMD cycles improve obese leukemic C57BL/6J mouse survival after vincristine treatment. (**a**) Experimental scheme, (**b**) body weight (gr) and (**c**) survival curve of periodical FMD in obese ALL murine model (*n* = 20) (*p* < 0.01 ** vs. HFD, ++ vs. FMD). A total of 20 diet-induced obese C57BL/6J mice were implanted with 1 × 10^4^ GFP-expressing BCR-ABL syngeneic leukemia cancer cells on day 0. Mice were randomized into groups of 5 mice with the following conditions: obese mice remaining on high-fat diet without vincristine (HFD) and with vincristine (HFD + VC) and obese mice receiving intermittent FMD without vincristine (HFD + FMD) and with vincristine (HFD + FMD + VC). VC (0.5 mg/kg/wk × 4 wks) was delivered I.P. beginning on day 7 (*n* = 5/group). (**d**,**e**) FACS analyses and representative histograms of GFP+ ALL cells in spleens taken from obese mice in the third week of treatment with or without FMD and VC (*n* = 3/group). Data are expressed as mean ± s.e.m. * *p* < 0.05, ** *p* < 0.01, one-way ANOVA.

C57BL/6J (20 weeks old) obese mice raised on a high-fat (60%) diet were implanted with GFP-expressing BCR-ABL syngeneic leukemia cancer cells [16]. Seven days post-implantation, obese mice were randomized into four groups: high-fat diet (HFD), HFD + FMD (FMD 3–4 days a week for 4 weeks), HFD mice treated with vincristine and HFD mice treated with FMD plus VC (Figure 1a). Mice were routinely examined for tumor progression and body weight (Figure 1b). FMD alone did not improve ALL survival but did improve survival in combination with VC; 40% of HFD+FMD+VC mice showed long-term cancer-free survival versus 20% in the HFD+VC group and none in the HFD or HFD+FMD only groups (Figure 1c). In a separate experiment, flow cytometric analyses showed a major reduction of GFP^+^ tumor cells in HFD+FMD+VC vs. HFD+VC spleens isolated from obese mice sacrificed after three weeks of treatment (Figure 1d,e). 

### 3.2. FMD Cycles in Combination with Vincristine Promote Cancer-Free Survival in a Normal-Weight Syngeneic Mouse Model of Pre-B-ALL

**Figure 2:**
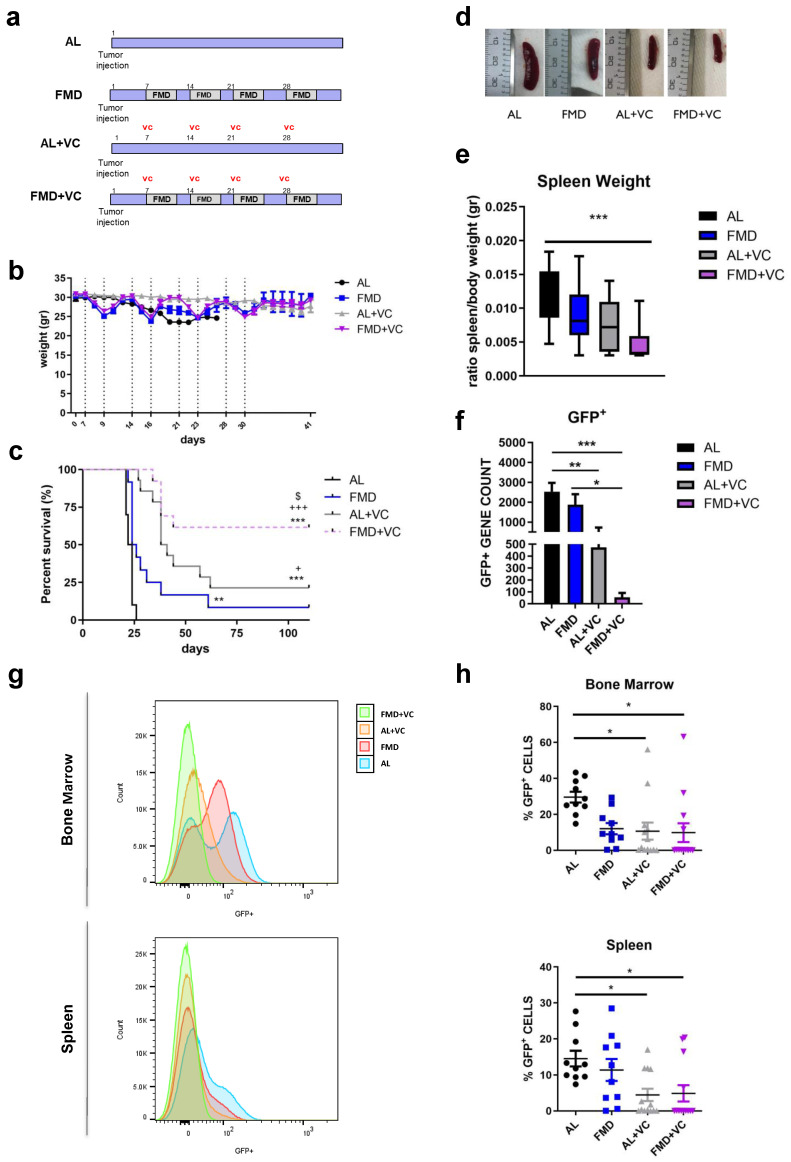
Effect of fasting-mimicking diet (FMD) in combination with vincristine in an in vivo model of BCR-ABL B-ALL. (**a**) Experimental scheme, (**b**) body weight (gr) and (**c**) survival curve of periodical FMD and vincristine in ALL in vivo model. Dashed vertical lines denote the FMD cycle. A total of 50 mice C57BL/6J (20 weeks old) were injected via retro-orbital injection with 1 × 10^4^ GFP-expressing BCR-ABL syngeneic leukemia cancer cells/mice. One week later, the mice were divided into 4 groups: ad lib+ vehicle (AL *n* = 10), fasting-mimicking diet (FMD *n* = 12) 4 cycles of 3 days + vehicle, ad lib+ chemo drugs (vincristine (AL + VC *n* = 14)) I.P. 0.5 mg/kg once a week and FMD +VC (*n* = 14) (*p* < 0.01 **, *p* < 0.001 *** vs. AL: *p* < 0.05 + *p* < 0.001 +++ vs. FMD: *p* < 0.05 $ vs. AL + VC). (**d**) Representative spleen picture, (**e**) spleen weight (gr) and (**f**) GFP+ RNA expression data (*n* = 6/group). (**g**) Representative histograms and (**h**) FACS analyses quantification for GFP+ tumor cells in bone marrow and spleen. Data are expressed as mean ± s.e.m. * *p* < 0.05, ** *p* < 0.01, *** *p* < 0.001, one-way ANOVA.

In a second in vivo model, we investigated the effect of periodic cycles of FMD + VC to treat ALL in mice maintained on a standard chow diet and of normal weight. C57BL/6J mice were injected with GFP-expressing BCR-ABL syngeneic leukemia cancer cells and one week later divided into four groups: regular chow ad libitum (ad lib+ vehicle (AL *n* = 10)), FMD (*n* = 12) + vehicle, ad lib+ vincristine (AL + VC *n* = 14) and FMD +VC (*n* = 14) (Figure 2a). Mice on FMD cycles exhibited acute weight loss each cycle, which was reversed during refeeding (Figure 2b). FMD alone did not improve median survival compared to the untreated standard chow group (25 vs. 23 days, *p* = *n*.s.) but did result in a significant improvement in overall survival (Figure 2c). Over half of the FMD + VC group survived more than 110 days without any leukemia symptoms, indicating a major improvement in cancer-free survival compared to the AL + VC group (*p* < 0.05 vs. AL + VC) (Figure 2c). In agreement with this finding, FMD + VC mice exhibited the smallest spleen size, weight, and GFP expression by qPCR (Figure 2d–f). Flow cytometry confirmed low numbers of GFP^+^ tumor cells in the bone marrow and spleen. On the day of autopsy, a reduction of ALL cells in both organs and a complete absence of tumor cells in the FMD + VC mice with long survival was shown (Figure 2g,h; Supplementary Figure S1). 

### 3.3. In Vitro Short-Term Starvation Reduces Cell Proliferation of Mouse and Human Leukemia Cells and Inhibits Autophagy

**Figure 3:**
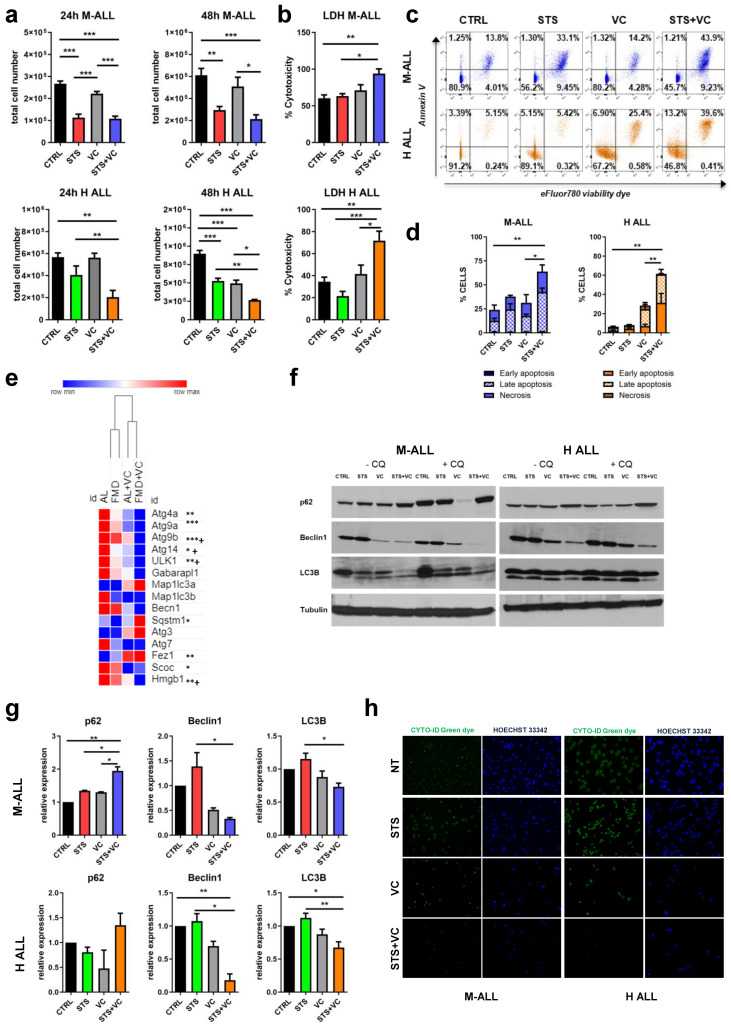
Effects of STS in combination with vincristine on mouse and human ALL cell lines. (**a**) Cell count and (**b**) LDH release of mouse and human ALL tumor cells were cultured in low-glucose (0.5 g/L) and 2% serum (in vitro STS) or in a standard glucose and 10% serum (control CTRL) medium ± vincristine 5 nM for 24 and 48 h. (**c**) FACS analyses of AnnexinV and eFluor780 viability dye of M-ALL and H-ALL tumor cell line and (**d**) percentage data quantification of cells in early and late apoptosis and necrosis. (**e**) Heatmap displaying autophagy gene expression in spleen tissue from AL, FMD, AL + VC and FMD +VC mouse (*n* = 6/each group) * *p* < 0.05, ** *p* < 0.01, *** *p* < 0.001 vs. AL and + *p* < 0.05 vs. VC. (**f**) Protein analyses of LC3B, beclin1, p62 and tubulin with or without chloroquine (CQ) 100 µM in M-ALL and H-ALL cell line. (**g**) Relative protein quantification performed by densitometric analysis using ImageJ64 software. Data are expressed as mean ± s.e.m. * *p* < 0.05, ** *p* < 0.01, *** *p* < 0.001, one-way ANOVA (*n* ≥ 3). (**h**) Cyto-ID green dye and Hoechst blue staining of M-ALL and H-ALL cells in normal or in STS media with or without VC 5 nM for 48 h.

We investigated the in vitro effects of short-term starvation (STS) on mouse (8093, M-ALL) and human (BV173, H-ALL) ALL cell lines grown under normal culture media (CTRL) or fasting-mimicking condition (STS medium with 0.5 gr/L glucose and 2% FBS) [17], with or without VC 5 nM. The STS medium reduced M-ALL and H-ALL proliferation (Figure 3a). STS in combination with VC decreased the cell number of both M-ALL and H-ALL cells by 70% by 48 h compared to the control medium. STS + VC also induced cell death as shown by a significant increase in lactate dehydrogenase (LDH) release (Figure 3b) and AnnexinV/eFluor780 viability dye staining. Compared to VC alone, 48 h of FMD + VC treatment increased apoptotic and necrotic M-ALL cells from 17.58 ± 2.18% to 42.32 ± 5.38% and H-ALL cells from 24.60 ± 4.44% to 60.66 ± 5.44% (Figure 3c,d). These analyses confirmed the effect of STS in enhancing VC-mediated cell death.

To dissect the molecular mechanism responsible for the effect of FMD + VC on cancer-free survival, we performed RNA-seq analyses on spleen tissue isolated from BCR-ABL B-ALL mice at the end of the treatment (Figure 3e). Our data revealed in the FMD + VC group the downregulation of genes involved in the initiation of autophagy, including ULK1, Atg4, Atg9a and Atg9b, and the up-regulation of autophagy genes such as Sqstm1 (p62) and FEZ1 in comparison to AL and AL + VC mice. FEZ1 is a negative regulator of LC3 lipidation [18], and when overexpressed, inhibits p62 degradation possibly through sequestration of ULK1. However, because these gene expression levels might be confounded by a higher splenic ALL cell burden in the AL and FMD groups than those treated with VC (Figure 2f), comparison between the first two lanes of Figure 3e have limited value, whereas the third and fourth lane can be compared.

Consistent with this result, protein expression analyses of autophagic markers indicate downregulation of LC3B and beclin1 in spleen tissue from the FMD + VC group (Supplementary Figure S2a,b). 

Autophagy plays a dual role in cancer pathogenesis: it can either promote or suppress tumor growth and survival [19,20,21]. To better understand the effect of STS and VC on autophagy, we analyzed key autophagic markers by Western blot in mouse and human ALL cells (Figure 3f,g). In agreement with the RNAseq analysis of spleen cells, STS + VC reduced expression of LC3B and beclin1 and increased the expression of the autophagy inhibitor p62. To quantify autophagy flux and LC3B turnover, chloroquine (CQ, 100 µM) was added to some samples 3–6 h before preparing the lysate. LC3B levels were not increased by chloroquine under FMD + VC conditions, indicating that autophagic flux is decreased.

These results were confirmed by Cyto-ID green, which showed strong staining in ALL cells that was reduced by VC and reduced further by STS + VC (Figure 3h). These results indicate that autophagy is highly active in ALL cells, but it is inhibited by FMD + VC. 

### 3.4. In Vivo Synergistic Effect of Autophagy Inhibition and FMD Treatment

**Figure 4:**
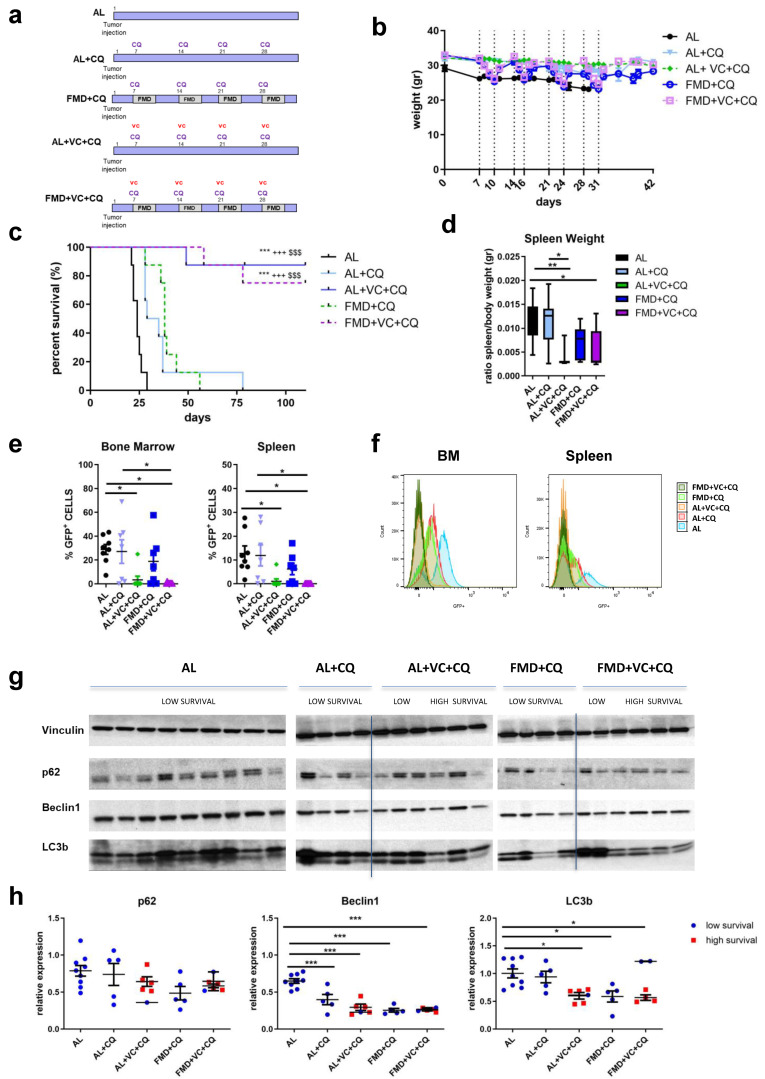
Effect of FMD in combination with vincristine and chloroquine in an in vivo model of BCR-ABL B-ALL. (**a**) Experimental scheme, (**b**) body weight (gr) and (**c**) survival curve. Dashed vertical lines denote the FMD cycle. A total of 40 mice C57BL/6J were injected via retro-orbital injection with 1 × 10^4^ GFP-expressing BCR-ABL syngeneic leukemia cancer cells/mice. One week later, the mice were divided into 5 groups: ad lib+ vehicle (AL), ad lib+ chloroquine once a week I.P. 50 mg/kg/day (AL + CQ *n* = 8), fasting-mimicking diet (FMD + CQ *n* = 8) 4 cycles (3–4 days) + CQ, ad lib+ VC + CQ once a week (AL + VC + CQ *n* = 8) and FMD +VC + CQ (*n* = 8) (*p* < 0.001 *** vs. AL, +++ vs. AL + CQ, $$$ vs. FMD + CQ). (**d**) spleen weight (gr). (**e**) FACS analyses representative histograms and (**f**) quantification for GFP+ tumor cells in bone marrow and spleen. (**g**) Protein analyses of LC3B, beclin1, p62 and vinculin in mouse spleen extract and (**h**) relative protein quantification performed by densitometric analysis using ImageJ64 software. Data are expressed as mean ± s.e.m. * *p* < 0.05, ** *p* < 0.01, *** *p* < 0.001, one-way ANOVA.

To investigate whether autophagy inhibition may enhance or mediate the effects of fasting/FMD and VC against ALL progression in vivo, a syngeneic mouse model of BCR-ABL was treated with the autophagy inhibitor chloroquine in combination with an ad lib diet or FMD ± VC (*n* = 8/groups) (Figure 4a). Body weight was monitored during the treatment (Figure 4b). Weekly co-treatment with CQ and vincristine resulted in a dramatic increase of long-term survival in ad lib mice, which was not increased further by FMD treatment (Figure 4c). The combined VC + CQ treatment reduced the size of the spleen (Figure 4d), as well as detectible GFP+ tumor cells, irrespective of FMD treatment (Figure 4e,f). Protein expression of LC3B, beclin1 and p62 in terminal sacrificed mouse spleen tissue confirmed the effect of either CQ + VC or FMD + VC in inhibiting autophagy (Figure 4g,h). These findings indicate that ALL cells are protected from VC treatment by an autophagy-dependent mechanism, which is reversed/blocked by co-treatment with either FMD or the autophagy inhibitor CQ.

### 3.5. Reduced Expression of Autophagy Genes Enhances VC-Dependent Apoptosis in ALL Cells 

**Figure 5:**
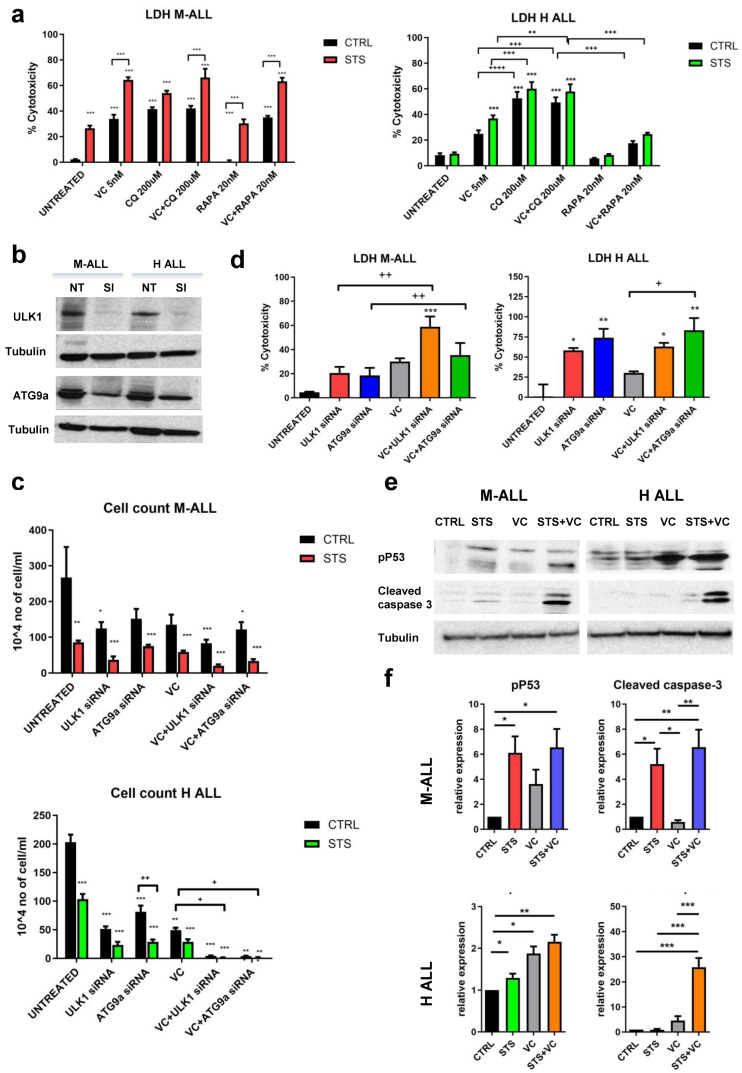
In vitro effects of STS in combination with vincristine on autophagy, cell death, and apoptosis. (**a**) LDH release of M-ALL and H-ALL cells cultured in STS or CTRL medium ± VC 5 nM for 48 h with or without chloroquine CQ 200 mM or rapamycin (RAPA) 20 nM. (**b**) Protein analyses of ULK1, ATG9a and tubulin after 48 h of transfection with the specific siRNA (Si) in normal medium. (**c**) Cell count and (**d**) LDH release of M-ALL and H-ALL after transfection with ULK1 siRNA (30 pM) or ATG9a (30 pM) siRNA with or without VC. (**e**) Western blot analyses and (**f**) relative protein quantification of phosphorylated p53, cleaved caspase 3 and tubulin in M-ALL and H-ALL cells (*n* ≥ 3). Data are expressed as mean ± s.e.m. * *p* < 0.05, ** *p* < 0.01, *** *p* < 0.001, + *p* < 0.05, ++ *p* < 0.01, +++ *p* < 0.001, ++++ *p* < 0.0001 one-way ANOVA.

To investigate the mechanisms of STS/VC-dependent ALL death, the viability of M-ALL and H-ALL cells grown in a normal or STS medium with CQ was measured. CQ treatment caused cytotoxicity of M-ALL and H-ALL cells cultured in either a normal or STS medium. Rapamycin, an autophagy activator, did not significantly affect cytotoxicity, though it caused a trend to reduce VC cytotoxicity in H-ALL (Figure 5a). CQ alone induced maximal toxicity in H-ALL cells, with or without VC, and this was potentiated in STS. 

Similar patterns were observed when we inhibited autophagy by gene knockdown. Autophagy genes ULK1 and ATG9 were knocked down in M-ALL and H-ALL cell lines with two specific siRNA (ULK1-siRNA and ATG9-siRNA). We confirmed that the expression of ULK1 and ATG9 proteins was inhibited by siRNA transfection (Figure 5b). Knockdown of ULK1 and ATG9a inhibited cell proliferation (Figure 5c) and promoted cell death, resulting in increased LDH release (Figure 5d). These results confirm that the inhibition of autophagy inhibits the growth and survival of leukemia cells. 

To validate the role of STS + VC in activating apoptosis, we measured the effect of the combined treatment on the phosphorylated form of p53 (Ser15) and the activation of the pro-apoptotic enzyme caspase-3. Activation of p53 by phosphorylation can lead to either cell cycle arrest or apoptosis. Protein analyses of phospho-p53 and cleaved caspase-3 showed a potentiating effect of STS on the toxicity of VC, particularly in H-ALL (Figure 5e,f). Taken together, these data suggest that fasting/FMD plus VC induce apoptosis in murine and human B-ALL cells in part by an autophagy- and p53-dependent mechanism.

### 3.6. Effect of FMD + VC on Immune Response against BCR-ABL B-ALL Cancer

**Figure 6:**
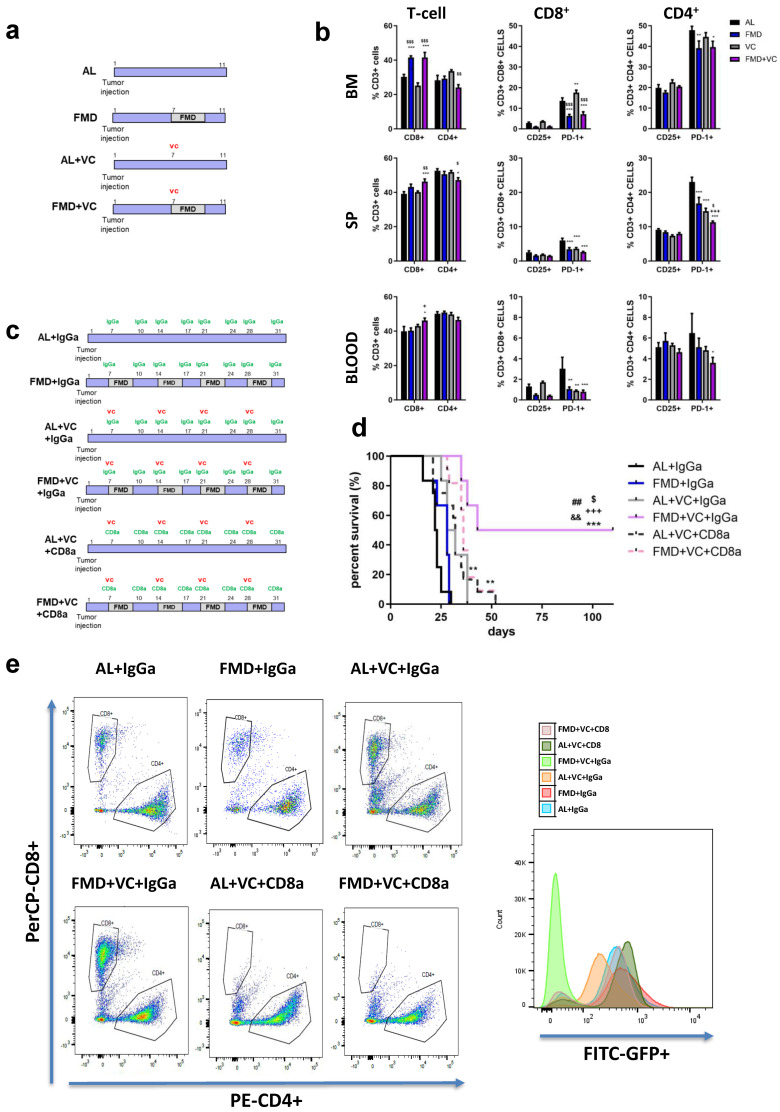
Effect of FMD in combination with vincristine on the immune response. (**a**) Experimental scheme of one cycle of FMD 3 days and vincristine in ALL in vivo model (*n* = 24). (**b**) FACS analyses quantification for CD3+CD4+, CD3+CD8+, CD3+CD4+PD-1+, CD3+CD4+CD25+, CD3+CD8+PD-1+ and CD3+CD8+CD25+ in bone marrow, spleen and blood. Data are expressed as mean ± s.e.m. * *p* < 0.05, ** *p* < 0.01, *** *p* < 0.001 vs. CTRL, + *p* < 0.05, ++ *p* < 0.01, ++ *p*< 0.001 vs. FMD, $ *p* < 0.05 vs. VC one-way ANOVA. (**c**) Experimental scheme and (**d**) survival curve of periodical FMD and VC in ALL in CD8+ in vivo depletion model. A total of 54 mice C57BL/6J (20 weeks old) were injected via retro-orbital injection with 1 × 10^4^ GFP-expressing BCR-ABL syngeneic leukemia cancer cells/mice. One week later, the mice were divided into 6 groups: ad lib+ IgGa (AL + IgGa *n* = 12), ad lib+ VC once a week (AL + VC + IgGA *n* = 6), ad lib+ VC+ CD8a (AL + VC + CD8a *n* = 12), FMD (3 days) + IgGa (*n* = 6), FMD +VC + IgGa (*n* = 6) and FMD + VC + CD8a (*n* = 12) (*p* < 0.001 *** vs. AL + IgGa, +++ vs. FMD + IgGa, $ *p* < 0.05 vs. VC + IgGa, *p* < 0.01 ## vs. VC + CD8a, && vs. FMD + VC + CD8a). (**e**) Representative FACS plots for CD3+CD4+ and CD3+CD8+ and histograms for GFP+ cancer cells in the bone marrow.

T cells can play an important role in the anti-tumor immune response. We previously showed that T cells mediate part of the effects of FMD against melanoma and breast cancer mouse models [22]. To determine whether FMD + VC may promote immune-dependent killing of B-ALL cells, we treated ALL mice with a single FMD cycle (*n* = 24 mice) and collected bone marrow, spleen and blood for flow cytometry analyses of the following T cell markers: CD3+CD4+, CD3+CD8+, CD4+CD25+, CD4+PD-1+, CD8+CD25+ and CD8+PD-1+ (Figure 6a,b). One cycle of FMD + VC shifted T cell populations toward anti-cancer immunity, increasing the pool of CD3+CD8+ in all three compartments, while reducing the pool of CD3+CD4+ cells in the bone marrow and spleen. Moreover FMD+VC significantly reduced the pool of both CD4+PD-1+ and CD8+PD-1+ cells (Figure 6b).

To test whether CD3+CD8+ T cells were required for FMD-dependent sensitization of leukemia cells to VC, we selectively depleted CD8+ T lymphocytes using a neutralizing monoclonal antibody (αCD8) or an IgGa (control) (Figure 6c,e). FMD + VC + IgGa control mice displayed the best outcome, with half of the mice showing long-term survival. However, CD8a treatment reversed this survival benefit (Figure 6d), indicating that the effects of FMD + VC are mediated at least in part by T cells. 

## 4. Discussion

FMDs have the potential to enhance the efficacy of a wide variety of cancer treatments, weakening cancer cells by a process we termed differential stress sensitization (DSS) while strengthening normal cells by a response termed differential stress resistance (DSR) [11,17,23,24,25]. The effects of fasting/FMD in inducing DSS in both in vitro and in vivo models were previously shown to be mediated, in part, by the reduction of circulating IGF-1 and glucose levels [11,17,23,24]. In a mouse leukemia model, fasting alone reversed the progression of both B and T cell ALL but did not affect acute myeloid leukemia (AML) [26]. 

Here we show that cycles of FMD induce significant anti-leukemia efficacy and cancer-free survival when combined with vincristine, in part by activating T-cell-dependent anti-cancer effects. Fasting/FMD alone causes a trend for increasing autophagy, but when fasting/FMD is combined with vincristine, a significant and consistent downregulation of autophagy markers is observed. This role of autophagy in the FMD/VC-dependent toxicity to ALL cells is confirmed by the effect of the combination of vincristine with the autophagy inhibitor chloroquine, which also promotes p53 modulation, apoptosis and cancer-free survival in agreement with the established role of p53 in mediating cell death in AML and in solid malignancies [27,28]. 

Emerging data indicate that autophagy is a major contributor to chemotherapy resistance in AML, CLL, multiple myeloma, lymphoma and in nonhematologic cancers. Inactivation of autophagy by deletion of *Atg5* or *Atg7* prolonged survival in an AML mouse model. Furthermore, *Atg7*-deficient mice showed less chemoresistance to cytarabine treatment [29]. Pharmacological inhibition of ULK-1 when combined with TKI treatment reduces growth of a CML cell line and patient-derived xenografted CML cells in mouse models [30]. Thus, autophagy inhibition may represent an important therapeutic strategy against many malignancies [19,20,31,32,33,34,35]. The only inhibitors approved by the FDA are the antimalaria drug chloroquine and its derivative hydroxychloroquine (HCQ), which suppress autophagy by blocking autophagosome fusion and degradation in the final steps of autophagy. Both CQ and HCQ have been investigated in preclinical studies and in clinical trials in combination with chemotherapy, radiation therapy or other targeted therapies, and which have shown evidence of enhanced antitumor activity caused by these combination [19].

Notably, CQ alone was able to delay ALL growth but caused only a minor effect on the survival of mice (Figure 4 and Figure 5). CQ disrupts the lysosomal functions, leading to cell death, and enhances VC efficacy compared with single-drug administration.

This role of autophagy in acting as an escape pathway in ALL cells treated with vincristine, which results in cell death when blocked by FMD, is in agreement with our recent study in which ER+ breast cancer cells treated with estrogen signaling and cdk4/6 inhibitors were killed by the effect of fasting/FMD cycles in blocking an escape based on insulin, leptin and IGF-1 signaling [36]. It is also in agreement with the effect of fasting/FMD in forcing the activation of PI3K-AKT and mTOR-dependent escape pathways, which resulted in the death of triple negative breast cancer cells when blocked [37]. Similarly, here, the toxicity of vincristine was limited by an autophagy-dependent escape mechanism, which was blocked by either FMD or chloroquine. However, it is important to point out that autophagy-inhibiting drugs are likely to have a more specific toxic effect on a limited range of cancer cell line, while fasting/FMD cycles instead appear to render many therapies more effective not only against a range of blood cancers but also against most solid tumors tested. Thus, the mechanism of action of FMD involves forcing many cancer cell types to reduce the number of redundant pathways that prevent cell death by generating energy or providing building blocks for the synthesis of DNA, proteins etc. These FMD-activated escape pathways can then be targeted by drugs, which in a number of mouse models, lead to tumor regression or cancer-free survival. The combination of FMD and these targeted drugs is likely to recruit immune cells by causing damage, including oxidative damage and DNA mutation in cancer cells. This may explain why FMD cycles are often effective against both immunocompetent and immunodeficient mouse cancer models, although blocking T cell function often reduces their efficacy.

**Figure 7:**
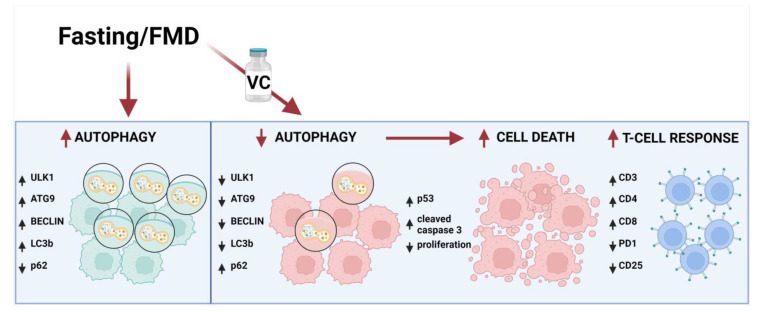
Schematic representation of FMD’s effect in combination with vincristine in leukemia cells. The effect of fasting or FMD alone on autophagy markers are based on the results from this study or the literature, but in the presence of VC, the autophagy pathway is inhibited by downregulation of Ulk1, ATG9, Beclin1 and LC3b and upregulation of p62. Consequently, apoptosis is activated by induction of p53 and cleaved caspase 3, leading to leukemic cancer cell death and increased T cell immune response against leukemia cells by activation of CD3, CD4 and CD8 expression and inhibition of PD-1 and CD25.

In a previous study on a murine model of breast and melanoma cancer, we demonstrated that FMD in combination with chemotherapy was able to stimulate T-cell-dependent cytotoxicity against tumor cells, promoting the infiltration of CD3+CD8+ TILs and decrease of Tregs at the tumor site; an effect partially mediated by the downregulation of heme oxygenase-1 [22]. In this study, FACS analyses showed that mice treated with FMD + VC had a higher presence of CD3+CD8+ cells and a reduction of CD3+CD4+ cells, together with a lower pool of PD-1+ T cells in the bone marrow, spleen and blood. Thus, the combination of FMD + VC generated changes commonly associated with immunotherapy and consistent with the role of the immune responses in cancer-free survival. In fact, the in vivo depletion of CD8+ cells reversed the effect of FMD + VC on cancer-free survival (Figure 6). Thus, this study, together with our previous work [22], supports the role of fasting/FMDs in inhibiting autophagy and boosting immune response in combination with chemotherapy, which result in the killing of leukemia cells (Figure 7).

Different studies have shown a relationship between the immune system and autophagy [38]. In a murine metastatic liver tumor model, a combination of IL-2 with CQ increased long-term survival and enhanced immune cell proliferation and infiltration in the liver and spleen [39]. A clinical trial (NCT01550367) is currently testing the combination of HCQ and IL-2 as a treatment in patients with metastatic renal cell carcinoma.

In mouse melanoma and mouse and human ovarian cancer, it has been shown that blocking the PD-L1/PD-1 axis via anti-PD1 or anti-PD-L1 antibodies can trigger autophagy in tumor cells, and when coupled with autophagy inhibitors, enhance the response [40]. Moreover, different papers have shown that blocking autophagy recruits different immune cells in the tumor environment, promoting cancer regression [41,42,43]. 

Fasting/FMD and other dietary restrictions have also been tested clinically in a number of clinical trials. In a prospective, nonrandomized, controlled trial of 40 patients, the potential benefits of caloric restriction were shown (The Improving Diet and Exercise in ALL (IDEAL)) in the efficacy of chemotherapy in patients newly diagnosed with B-ALL. This intervention resulted in a low minimal residual disease risk, high-circulating adiponectin and low insulin resistance [44]. In a randomized controlled study of 131 patients with HER2-negative early-stage breast cancer, FMD cycles significantly enhanced the effects of neoadjuvant chemotherapy on the radiological and pathological tumor response [45]. A short-term fasting-mimicking diet was also well tolerated during chemotherapy in patients with ovarian cancers and appeared to improve quality of life and fatigue [46,47,48]. In conclusion, FMD cycles have high potential to be effective in increasing the toxicity of a range of therapies against ALL and other blood cancers and should be tested in randomized clinical trials, especially in combination with immunotherapy and low toxicity cancer therapies.

In summary, we present a new strategy for improving leukemia treatment by combining FMD with chemotherapy to promote the killing of ALL cells in part by an immune-dependent mechanism. Fasting/FMD has been shown to reduce chemotherapy-associated toxicity in pre-clinical and clinical studies [47,49,50] and thus represents a safe and potentially effective treatment adjunct for leukemia patients which should be tested clinically. 

## 5. Conclusions

FMD acts synergistically with vincristine to deplete B-ALL cells in mice. The beneficial effects of FMD on B-ALL progression and survival depend on autophagy inhibition and activation of anti-cancer immunity. The use of the autophagy inhibitor CQ can potentiate the effect of vincristine with or without fasting conditions. However, the use of autophagy inhibitors in cancer therapy could be challenging since, unlike FMD cycles, they may be effective only against a portion of cancers while also causing more side effects. These preclinical data provide proof of principle evidence for clinical trials testing the efficacy of the combination fasting/FMD with various therapies, including chemotherapy and immunotherapy.

## Data Availability

All the data supporting the findings of this study are available within the article and its supplementary information files and from the corresponding author upon reasonable request.

## Supplementary Materials

The following supporting information can be downloaded at: https://www.mdpi.com/article/10.3390/cancers15245870/s1, Table S1. Mouse FMD ingredients; Figure S1. Flow cytometry gating strategy to identify GFP+ BCR/ABL leukemia cells in the bone marrow and spleen; Figure S2. protein expression analyses of autophagic markers in-dicate downregulation of LC3B and beclin1 in spleen tissue from the FMD + VC group [51,52,53,54].
