# Supplementary material for: Fasting-Mimicking Diet Inhibits Autophagy and Synergizes with Chemotherapy to Promote T-Cell-Dependent Leukemia-Free Survival
Source: Cancers (Basel). 2023 Dec 16;15(24):5870. doi: 10.3390/cancers15245870 (PMC10741737; doi:10.3390/cancers15245870)
Supplement: Supplementary file 1 [file cancers-15-05870-s001.zip › cancers-2734302-supplementary.pdf]

Table S1. Mouse FMD ingredients

	Day 1 (g)	Days 2-3 (g)	Note
Broth mix	5.95	5.31	The blend ratio of 2 broth mixes is 1:1.<br>• Vegetable Broth Mix (#60017, ABCO Laboratories, Fairfield, CA): Ingredients: Maltodextrin, Salt, Autolyzed yeast extract, Natural Flavor, Dehydrated Vegetables (tomato, onion, celery, parsley, spinach, garlic, carrot), Potato Flour, Xanthan Gum, Spices, Extractive of Spice (including turmeric), Soy Lecithin. Not more than 2% Soybean Oil added as a processing aid.<br>• Chicken Flavor Broth Mix (#60016, ABCO Laboratories, Fairfield, CA): Ingredients: Maltodextrin, Salt, Autolyzed yeast extract, Natural Flavor, Dehydrated Vegetables (onion, celery, parsley, spinach, garlic, carrot), Potato Flour, Xanthan Gum, Spices, Extractive of Spice (including turmeric), Soy Lecithin. Not more than 2% Soybean Oil added as a processing aid.
EVOO	12.75	0	EVOO, Bertolli Extra virgin olive oil (Mizkan American, Inc, Mount Prospect, Illinois).
Essential fatty acid	0.21	0	Udo's Oil, 3*6*9 (Udo's Choice) Blend Ratio Ω3:6:9 (Omega-3, alpha-linolenic acid; Omega-6, linoleic acid; Omega-9 olieic acid) is 6:3:2.5. (Flora Incorporated, Lynden, Washington).
Vegetable mix	14.88	0	• Beet Root powder (RM10711, The Synergy Company, Moab, Utah) (2.86 g).<br>• Carrot Root powder (RM10437, The Synergy Company, Moab, Utah) (1.86 g).<br>• Collard Leaf powder (RM10114, The Synergy Company, Moab, Utah) (1.24 g).<br>• Kale Leaf powder (RM10115, The Synergy Company, Moab, Utah) (1.24 g).<br>• Nettle Leaf powder (RM10130, The Synergy Company, Moab, Utah) (1.24 g).<br>• Spinach Leaf powder (RM10747, The Synergy Company, Moab, Utah) (2.48 g).<br>• Tomato Fruit powder (RM10409, The Synergy Company, Moab, Utah) (2.48 g).<br>• Mitake Mushroom powder (ZNature Foods, West Palm Beach, FL) (1.86 g).
Glycerol	0	0.75	G9012, Sigma-Aldrich, St. Louis, Missouri.
Hydrogel (binding agent)	56.7	93.9	Clear H2O, Westbrook, Maine.
Fiber	5	0	Cellulose, #3425, Bio-Serv, Flemington, New Jersey.
Mineral (AIN-93G-MX)	3.5	0	Teklad, TD.94046, ENVIGO.
Vitamin (AIN-93-VX)	1	0	Teklad, TD.94047, ENVIGO
Sum	*100*	*100*	

Supplementary methods

Mouse FMD diet: The mouse version of the FMD is a 3-day regimen (49-50). All diet ingredients were thoroughly mixed and then blended together with heated hydrogel (ClearH2O, Maine) (Table S1). Mice on the FMD diet were fed 50% of the standard daily calorie intake on day 1 and 10% of normal daily calorie intake on days 2 to 3. All mice were supplied with fresh food during the morning hours (8am-10am). **Post-FMD refeeding:** after the end of the day 2-3 diet, mice were fed ad libitum regular chow for up to 4 days to regain body weight before the next FMD cycle.
Peroxidase conjugated IgG (Santa Cruz, CA, USA) was used as secondary antibody.

RNA-seq library preparation and data analysis

Total RNA was extracted from the spleen (n=6/each group) using RNeasy Mini kit (QIAGEN, Germantown, MD, USA) followed by on-column DNase I digestion as per manufacturer's instructions. RNA integrity was verified with a Bioanalyzer (Agilent Technologies, Santa Clara, CA, USA). RNA was quantified with the Qubit fluorometer (Invitrogen, Walthman, MA, USA), and 1 µg of purified RNA was utilized as starting material for library construction, which was carried out with the KAPA RNA HyperPrep Kit with RiboErase (KAPA BIOSYSTEM, Wilmington, MA, USA Cat#KK8560) as per manufacturer's instructions. Libraries were sequenced with an Illumina HiSeq 3000 system using 50-bp single-end reads.

The RNA-Seq reads are mapped to the mouse GRCm38 references using HISAT2 (2.1.0) [51] with tuned parameters determined in [52].

The script used to map reads is available at https://github.com/smangul1/recycle.RNA.seq/blob/master/benchmark_RNASeq_aligners/code/run.hisat2.tuned.sh. The resulting mapped reads of each sample are stored in a binary format file (.bam). Gene counts were obtained by counting the number of sequencing reads overlapping each of the genes (HTSeq v0.6.1) [53]. Gene annotations (ENSEMBL GRCh38, gtf format) were downloaded

from www.ensembl.org. The script htseq-count is supplied with the GTF file with gene models and a bam file containing the mapped reads. The script generates individual gene counts by examining the read overlap with the genes. We choose a conservative setting (--mode=intersection-strict) to handle reads overlapping more than one feature. Thus, reads overlapping several genes simultaneously are marked as reads with no feature and excluded from consideration. Gene counts from every sample are used to determine genes differentially expressed across the conditions (DeSeq2 with default parameters). DeSeq2 models read counts as a negative binomial distribution and uses shrinkage estimation for dispersions and fold changes [54]. Within-group variability (variability between replicates) is used to determine if the observed difference between the conditions may be attributed to the experimental conditions and not to the biological variance. FDR corrected p-values are used to control the number of false positives.

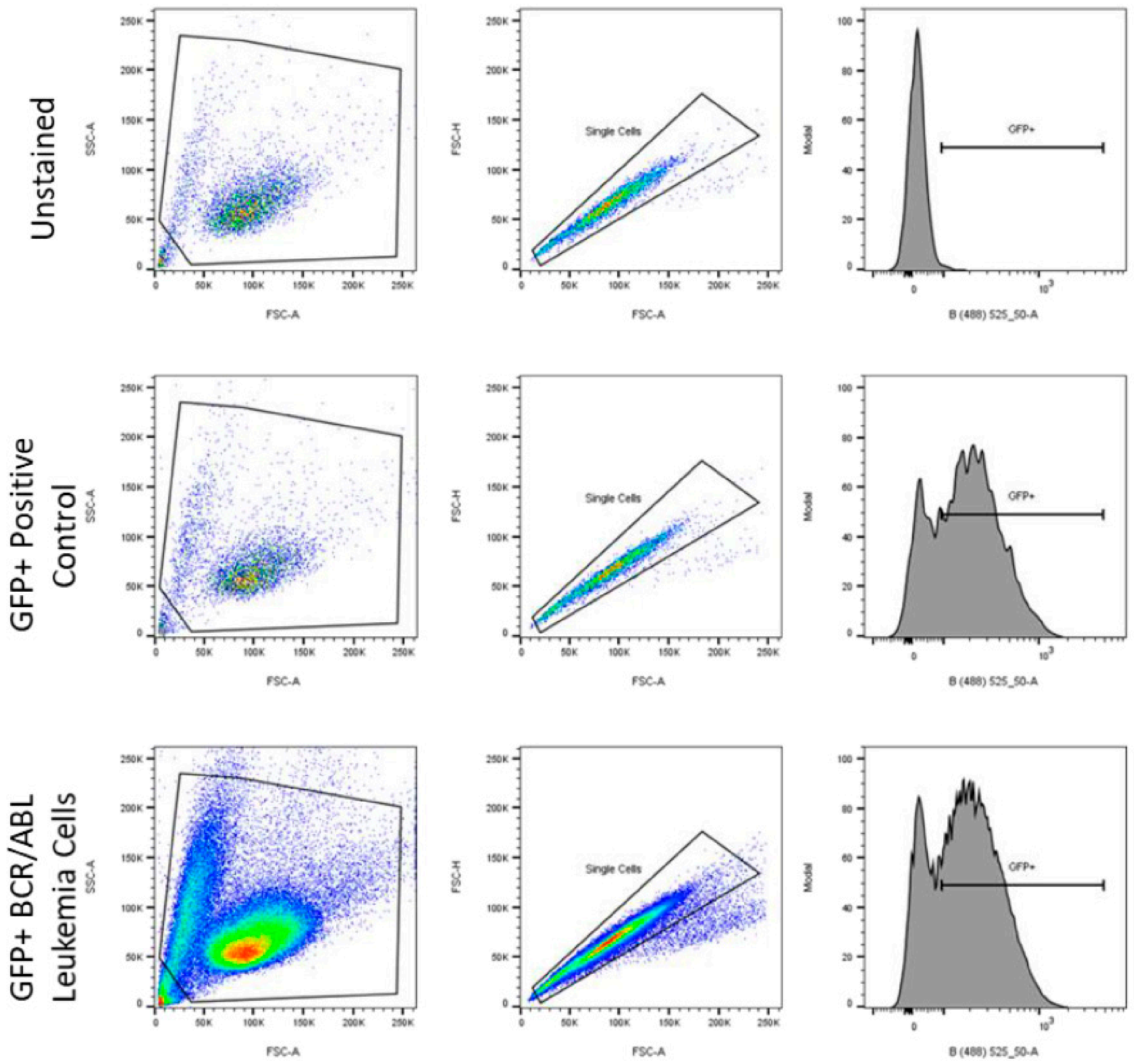

Figure S1. Flow cytometry confirmed low numbers of GFP+ tumor cells in the bone marrow and spleen.

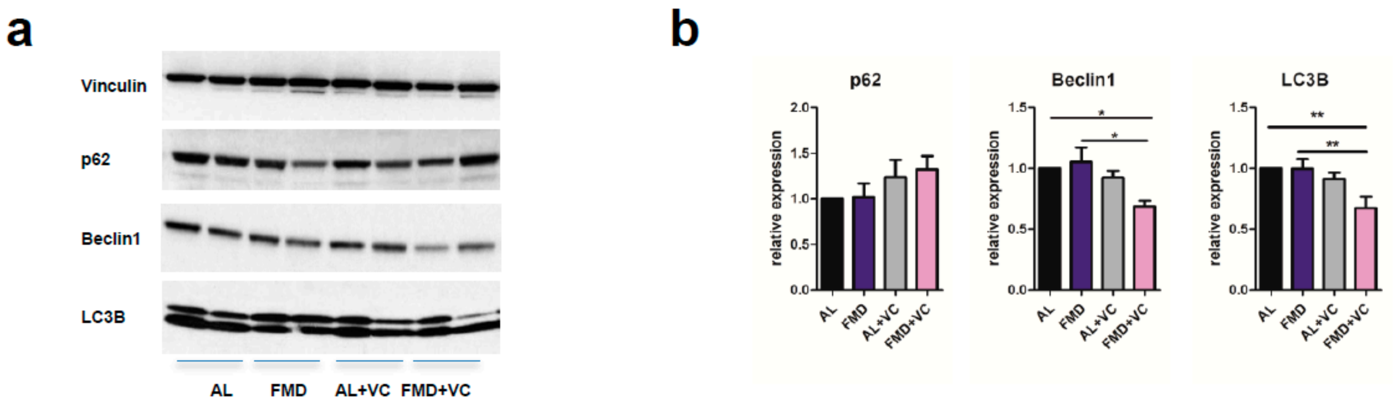

Figure S2. protein expression analyses of autophagic markers in-dicate downregulation of LC3B and beclin1 in spleen tissue from the FMD + VC group.
